# High-Density Plasmonic Nanoparticle Arrays Deposited on Nanoporous Anodic Alumina Templates for Optical Sensor Applications

**DOI:** 10.3390/nano9040531

**Published:** 2019-04-03

**Authors:** Uldis Malinovskis, Raimonds Poplausks, Donats Erts, Kerstin Ramser, Sigitas Tamulevičius, Asta Tamulevičienė, Yesong Gu, Juris Prikulis

**Affiliations:** 1 Institute of Chemical Physics, University of Latvia, 19 Raina Blvd., LV-1586 Riga, Latvia; uldis.malinovskis@lu.lv (U.M.); raimonds.poplausks@lu.lv (R.P.); donats.erts@lu.lv (D.E.); 2 Department of Engineering Sciences and Mathematics, Luleå University of Technology, SE-97187 Luleå, Sweden; kerstin.ramser@ltu.se; 3 Institute of Materials Science, Kaunas University of Technology, 59 K. Baršausko St., LT-51423 Kaunas, Lithuania; sigitas.tamulevicius@ktu.lt (S.T.); asta.tamuleviciene@ktu.lt (A.T.); 4 Department of Chemical and Materials Engineering, Tunghai University, Taichung 40704, Taiwan; yegu@thu.edu.tw

**Keywords:** porous anodic aluminum oxide, colloid deposition, plasmonics, nanoparticle arrays, hemoglobin, SERS

## Abstract

This study demonstrates a new, robust, and accessible deposition technique of metal nanoparticle arrays (NPAs), which uses nanoporous anodic alumina (NAA) as a template for capillary force-assisted convective colloid (40, 60, and 80 nm diameter Au) assembly. The NPA density and nanoparticle size can be independently tuned by the anodization conditions and colloid synthesis protocols. This enables production of non-touching variable-density NPAs with controllable gaps in the 20–60 nm range. The NPA nearest neighbor center distance in the present study was fixed to 100 nm by the choice of anodization protocol. The obtained Au NPAs have the resonant scattering maxima in the visible spectral range, with a refractometric sensitivity, which can be tuned by the variation of the array density. The thickness of the NAA layer in an Aluminum-NAA-NPA multilayer system enables further tuning of the resonance frequency and optimization for use with specific molecules, e.g., to avoid absorption bands. Applicability of the mentioned multilayers for colorimetric refractive index (RI) sensing is demonstrated. Their use as Surface-Enhanced Raman Scattering (SERS) substrates is tested using hemoglobin as a biological probe molecule.

## 1. Introduction

Noble metal nanoparticles support oscillations of free electrons, known as localized surface plasmon resonances (LSPR), with frequencies in the visible or near-infrared spectral range, which is sensitive to particle shape, environment [1], and interactions with other nanoparticles [2]. The LSPR is accompanied by (i) high intensity electromagnetic fields near the nanoparticle surface and (ii) a strong radiative scattering. The relationship between these two effects provides a useful and convenient method for optical far-field detection of local properties in the immediate surrounding of the nanoparticles. Arrays of plasmonic nanostructures express additional features in the scattered fields such as modified angular distribution [3] or reduced spectral peak width [4], which can be used, for example, to increase the sensitivity or resolution of the LSPR-based sensors [5,6]. Assemblies with narrow gaps between the individual metal nanoparticles can be of interest for electronic sensor readouts, which probe the resistivity of interlinked two-dimensional molecular–NPA networks [7] and are also expected to be efficient substrates for Surface-Enhanced Raman Scattering (SERS) [8,9]. Periodic NPAs can also form photonic crystals, which often are produced by close-packed colloid assemblies [10]. Arrays with some degree of disorder may exhibit new effects, such as phase shifts or change of polarization [11] that can be used for interferometric sensing of biomolecules [12,13]. However, reliable, reproducible, and cost-effective production of the nanoparticle array substrates for label-free sensor architectures remains challenging.

A viable route to satisfy the requirements of high particle density and scalability involves the use of self-organized materials such as nanoporous anodic alumina (NAA) also known as porous anodic aluminum oxide (PAAO). The NAA has been used for production of plasmonic nanoparticle arrays (NPA) for more than two decades [14]. Typically, NAA serves as a shadow mask for metal deposition [15,16,17,18] or pores are filled with metal electrochemically [9,15,19,20,21]. Some materials, for example germanium, can be filled in NAA pores using the supercritical fluid method [22]. In all cases, the nanoparticle size, shape and array density are determined by the NAA morphology and there are only very few options available for size tuning, i.e., pore widening and tilted sputtering. Additionally, the crystallinity of the nanoparticles obtained using these techniques is unpredictable; however, monocrystalline nanoparticles are of great interest for high performance plasmonic applications including LSPR sensors and SERS detection [23].

It should be noted that NAA without NPA has previously been used as base for optical sensor applications. The presence of an analyte inside the pores can modify the effective refractive index and change the optical thickness of the NAA layer, which can be registered by colorimetric and interferometric detection schemes [24,25]. Other potential uses of the NAA with Au NPA may also include catalysis [26].

Recently, sorting and ordering of polydisperse diamond and silver colloids on the NAA templates [27] was demonstrated without addressing their optical properties. In this work we present a new lithography-free, variable-density, non-touching NPA production method, where monodisperse Au nanoparticle colloids are deposited in arrays using the capillary force-assisted convective assembly. The NPAs can be produced at room temperature from prefabricated colloids that are well characterized and have the desired properties (material, size, shape, crystallinity, core–shell composition [28]) defined before deposition.

In comparison to other scalable and cost-competitive noble metal colloid deposition techniques for optical sensor applications [29,30,31,32], our method is truly lithography-free and produces isolated 2D particle arrays with small and controllable gaps (20–60 nm range).

As proof-of-concept experiments we studied the elastic light scattering from the obtained Au NPA for LSPR refractometric sensitivity and their applicability for SERS detection of hemoglobin. The actual resulting optical system is a new type of multilayer, which consists of a reflective aluminum substrate, an approximately 200–300 nm thick dielectric NAA spacer and an Au NPA. The array density can be varied from sparse individual nanoparticles to nearly 1:1 particle/pore filling ratio on the same sample, which can be of interest for development of lab-on-chip type devices. Well-established anodization protocols (0.3 M oxalic acid electrolyte at 40 V in the present study) can be used to produce templates for different size and type of nanoparticles.

## 2. Experimental

### 2.1. Materials

Pure Al foil (99.999%, 0.32 mm thickness, 100 × 100 mm sheets, Good Fellow) was used as the starting material for NAA synthesis. All acids and solvents were of analytical grade and used as received. Au colloids with three different nanoparticle diameters (40 nm, 60 nm and 80 nm, concentration OD 1, stabilized in a suspension of 0.1 mM PBS, Aldrich, Saint Louis, MO, USA) were used for NPA deposition. For Raman spectroscopic measurements bovine hemoglobin (MP Biomedicals, Solon, OH, USA, 100714) was dissolved in Phosphate buffered saline (PBS, pH 7.4, VWR, E504) at ratios 1 and 10 μg/mL.

### 2.2. Methods

A standard two step anodization protocol was used for the preparation of the NAA templates [14,27]. The Al sheets were cut in 9 × 20 mm pieces, degreased in acetone and electropolished in ice-cold perchloric acid—ethanol mixture (1 part 60% HClO_4_:4 parts 96% ethanol) at 15 V until a mirror-like surface finish was reached (approximately 5 min). The electropolished Al sheets were anodized at 15 °C temperature for 2 h in 0.3 M oxalic acid electrolyte under 40 V anodization potential. The anodic oxide layer was removed with a chromic–phosphoric acids solution (4% H_2_CrO_4_, 11% H_3_PO_4_) at 70 °C. NAA thin films with approximate thicknesses of 200–300 nm were produced during a 1 min long second anodization under the same conditions as the first one. After the second anodization step the samples were immersed in a 5% H_3_PO_4_ solution for 1 min long pore widening, washed with water, and immediately used as templates for colloid deposition.

Au colloids were deposited on the NAA surface by the dip-coating technique at withdrawal speeds $v_{w}$ of 0.1, 0.5 and 2 μm/s. Hb solutions for SERS measurements were placed between the Al-NAA-Au NPA substrate and a standard microscope slide. Array particle size and distribution were analyzed with a field emission scanning electron microscope (FE-SEM-4800, Hitachi, Tokyo, Japan) and ImageJ ver. 1.52a software [33]. Optical spectra were collected using an inverted microscope (Olympus IX 71, Tokyo, Japan), which was fiber coupled either to a miniature UV-VIS-NIR spectrometer (Ocean Optics USB4000, Largo, FL, USA) for elastic scattering or a Raman spectrometer (Shamrock 303i, Andor Technology, Belfast, UK), equipped with a 532 nm excitation DPPS laser (Laserglow Technologies, Toronto, ON, Canada). The SERS signal was guided through the bottom port and focused into an optical fiber connected to the Raman spectrometer. Reference spectra from polystyrene were recorded for frequency calibration. The Raman spectra were smoothed, and baseline corrected by an in house developed MATLAB script, following the articles [34,35]. Side illumination at 45° angle from a halogen lamp (THORLABS OSL1-EC, Newton, NJ, USA) was used as light source for the dark-field measurements. GNU Octave functions leasqr and corr were used to find fitting parameters of spectral peaks and to calculate correlation coefficients.

**Figure 1:**
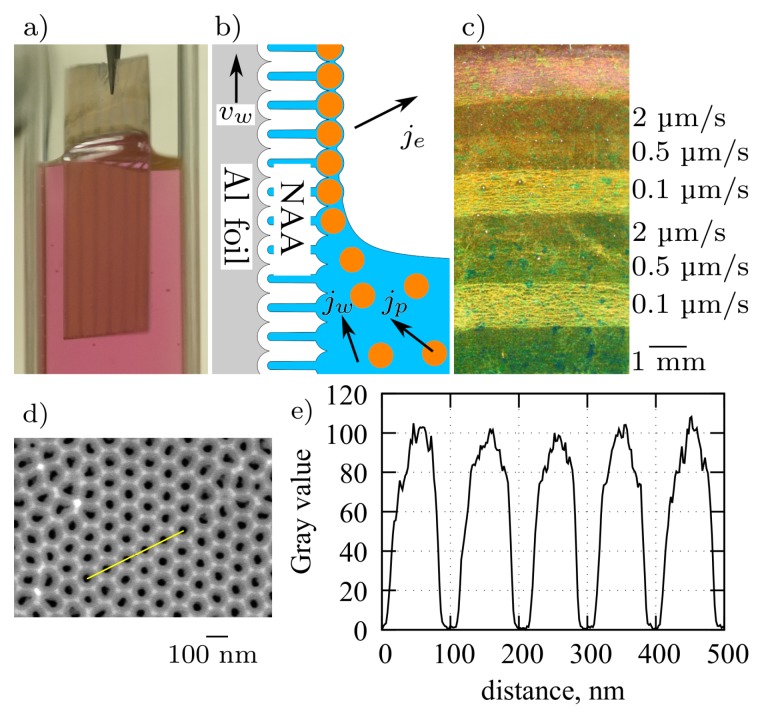
(**a**) Photograph of a NAA coated aluminum sheet during withdrawal from Au nanoparticle colloid. (**b**) Diagram of the convective nanoparticle assembly on a NAA surface. (**c**) Dark-field photograph of 80 nm Au NPAs on an Al-NAA substrate obtained by dip-coating with different withdrawal speeds. (**d**) Top view SEM image of NAA and (**e**) corresponding line profile from a region with well-ordered pores.

Figure 1a shows a photograph of a sample immersed in the Au colloid during withdrawal. The NAA is hydrophilic as indicated by the shape of meniscus. The principle of array assembly is shown in Figure 1b and is similar to the convective assembly of closely packed NPAs on smooth surfaces [36]. Briefly, the evaporation flux $j_{e}$ from the liquid film above the meniscus is compensated by a water flux $j_{w}$, which drives the particles towards the meniscus contact line. At higher withdrawal speeds ($v_{w}=2$ μm/s) the particle flux $j_{p}$ is insufficient and only a few particles per μm^2^ remain on the surface. The NAA enforces particle positioning in predefined locations on the surface, inside the funnel shaped pore openings [27]. At lower values of $v_{w}$ the particle density increases until nearly all allowed sites are occupied. For the type of above-mentioned colloids, it was found that $v_{w}$ = 0.1 μm/s is optimal for formation of sufficiently large monolayer array islands for optical analysis.

Figure 1c shows a photograph of an NAA sample after withdrawal at various speeds, where different intensity regions corresponding to different particle densities can be identified by unaided eye. For the NAA layer thickness dependent studies we used two effects, which occur concurrently during sample anodization: firstly, a gradual thickness change due to variations of temperature, current density, and electrolyte convection, and secondly, step-like thickness changes due to different chemical reaction rates on different crystallographic orientations of grains in the aluminum substrate [37]. The dip-coating procedure with different speeds was done using a custom built stepper-motor driven vertical translation stage placed on a passive vibration isolation system. The setup is fully automated and was typically run unattended (overnight) in ambient temperature. The humidity, which is an important parameter in capillary force-assisted particle assembly [38,39], was kept constant at a saturated level by the confined cuvette volume (Figure 1a) and by the continuous evaporation from the colloid surface. After withdrawal, the samples were left to dry at room temperature.

## 3. Results and Discussion

**Figure 2:**
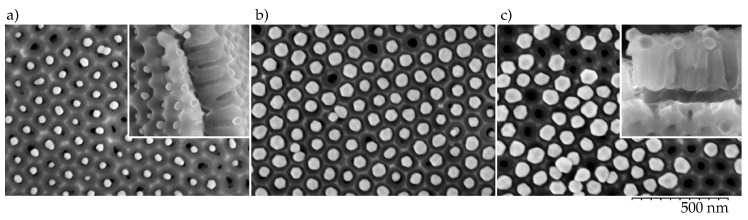
Top view SEM images of (**a**) 40 nm, (**b**) 60 nm, and (**c**) 80 nm Au nanoparticles on an oxalic acid electrolyte NAA surface. Insets show the sample cross-sections.

The highest-density particle arrays were formed by the dip-coating procedure when anodized aluminum plate was slowly withdrawn from the colloidal solution (0.1 µm/s). For a flat surface (without NAA) with a right balance of withdrawal velocity and particle flux towards the meniscus contact line, the nanoparticles would produce a closely packed array [36]. The asperities and pore walls of the NAA surface prevent close packing and nanoparticles are positioned above pore openings (Figure 2). The center-to-center distance of the nearest neighbors was 100 nm as determined by the distance between the pores in the NAA template (Figure 1d,e). The pore diameters were less than 40 nm, which confines the particles within the funnel cones and blocks their entrance into the pore depth, which is useful for distance control between the nanoparticles and the Al surface. Although some nanoparticles were misplaced or, occasionally, twin-particles could occupy the same site, stacking or bilayer growth was rarely observed in the steady deposition regions using the mentioned withdrawal speeds.

A general observation was that the 40 and 60 nm diameter particles yielded the best pore filling, whereas the particles with 80 nm diameter produced NPAs with more vacancies. The larger species protrude above the NAA surface and are more likely to be removed by the particle carrying liquid. This is in line with the size-dependent colloid sorting on NAA surface reported by [27], where NAA samples with much smaller pores (sulfuric acid anodization) were used to assemble sub-25 nm particle arrays.

**Figure 3:**
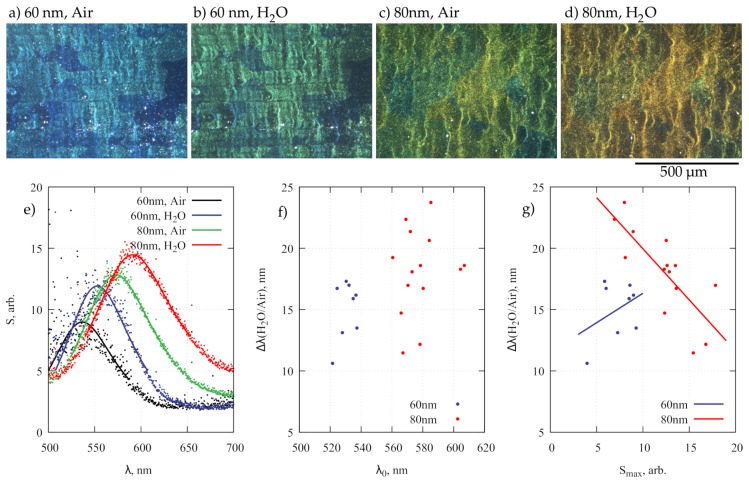
(**a**–**d**) Dark-field photographs of 60 and 80 nm NPAs on Al-NAA substrate in air and water illuminated by s-polarized white light. (**e**) Typical scattering spectra from samples (**a**–**d**), where the same spot was examined in air and water. (**f**) medium dependent (air/water) spectral shift of peak wavelength ∆λ as a function of peak wavelength in air, and (**g**) spectral shift dependence on scattering maximum amplitude $S_{max}$.

In practice, during sample withdrawal the contact line moved in a stick-slip fashion due to pinning effects [40] and concave islands of NPA were formed. Any surface defects such as traces from Al sheet rolling or inclusions protruding after electropolishing limited the continuous array assembly. However, the islands had a sufficient size (approximately 10 μm) to be observed in an optical microscope under dark-field illumination (Figure 3a–d). It should be noted that NAA with better hexagonal pore ordering in large domains can be achieved but this would require long anodization times, i.e., above 10 h [41,42]. Typically, ordered regions were relatively small and over distances comparable to the wavelength of the visible light the NPA looses periodicity. Hence, conditions for Bragg scattering or lattice resonances are absent and the averaged scattering spectra (Figure 3e) within the microscope collection area (approximately 20 μm diameter) resembled those of individual nanoparticles with a peak position near 550 nm and a width of about 80–100 nm [43] (Lorentzian fit with a width of 0.4 eV was used to find the maximum frequency in scattered spectra in Figure 3e). For comparison, much broader spectra in near-infrared region were observed for highly ordered close-packed (8 nm gaps) Au NPA of similar size [8].

There was little color change between high- and low-density regions as can be seen in (Figure 3a–d). A more noticeable spectral change is caused by the variation of the NAA layer thickness due to differences in the anodization rate on the surfaces of domains with different crystallographic orientations in the Al substrate (±15% variation of NAA thickness) [37] and gradients of current density within the anodization cell. The scattering from nanoparticles on the dominant (100) domains with a thicker NAA layer are redshifted relative to the less common grains of other orientations. The overall thickness variation of NAA after 1 minute long second anodization (see methods section) for samples used in this study was from 215 to 290 nm as determined from the SEM analysis of NAA cross-sections (insets in Figure 2).

One of the often envisioned plasmonic NPA applications is LSPR-based refractometric sensing. In an Al-NAA-Au NPA system, there are three factors, which may influence the RI sensitivity of scattered spectra: (i) particle size, which is known; (ii) NAA layer thickness, which can be estimated from the scattering maximum wavelength $\lambda_{0}$ in air; (iii) nanoparticle density, which is assumed to have monotonous relations to the scattering maximum intensity $S_{max}$. To study these effects, the sample was illuminated at a 45° angle with s-polarized white light and the scattered light S(λ) was collected within a 17.5° cone, normal to the sample surface (0.3 NA). Spectra from selected spots were first recorded in air, and then from the same spots in pure water with RI n=1.33 (Figure 3e). The mean values for the spectral shift ∆λ caused by a RI difference of ∆n = 0.33 were 15.0 and 18.1 nm (standard deviations 2.4 and 3.5 nm) for 60 and 80 nm diameter NPA, respectively. There was a large scatter in data when ∆λ was plotted as a function of $\lambda_{0}$, which depends on the NAA layer thickness *h* (Figure 3f). Assuming a linear relation between *h* and $\lambda_{0}$ (purely thin-film interference coloring [37]) the correlation coefficient ρ between the spectral shift and NAA layer thickness was small, only 0.40 (0.19) for 60 (80) nm diameter NPA. However, for the 80 nm diameter NPA, a more pronounced relation existed between the array density (estimated by the scattering maximum intensity $S_{max}$) and ∆λ (Figure 3g). The RI sensitivity in this case clearly decreased for larger values of $S_{max}$ with ρ=−0.78. For the 60 nm NPA the correlation between $S_{max}$ and ∆λ remained low ρ=0.38. The size-dependent relation between the RI sensitivity and array density requires detailed numerical simulations, which is beyond the scope of this article; however, it may present a route for NPA performance optimization [44]. The particles with larger diameters (80 nm) show better RI sensitivity, which agrees with the theoretical modeling [45]; however, the average sensitivity ∆λ per refractive index unit appears low (<75 nm/RIU). For the smallest nanoparticles (40 nm diameter) there were no scattering peaks in the visible spectral range that could be measured with sufficient signal to noise ratio, likely because for Au nanoparticles of this size absorption dominates over scattering [43].

**Figure 4:**
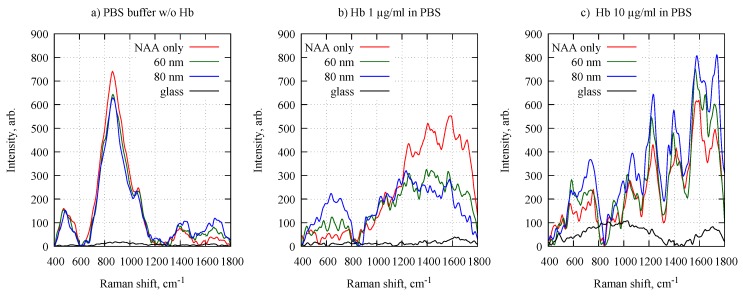
Typical Raman spectra of (**a**) PBS without hemoglobin and (**b**,**c**) different concentrations of hemoglobin diluted in PBS on a glass substrate, NAA substrate and NAA with 60 and 80 nm diameter Au NPAs.

Now we turn to the potential of applying the Al-NAA-Au NPA as substrates for SERS detection by using hemoglobin (Hb) as a test molecule. Figure 4 shows representative Raman spectra of different Hb concentrations in PBS solution on NAA substrates with 60 and 80 nm diameter NPA, glass surface, and NAA without any nanoparticles.

The obtained spectra from the three different NAA-based substrates (Figure 4) show clearly distinguishable bands of Hb in the core size region (1500–1650 cm${}^{-1}$), the pyrrole ring stretching region (1300–1400 cm${}^{-1}$), the methine C–H stretching region (1200–1300 cm${}^{-1}$), and in the low-wavenumber region (600–1200 cm${}^{-1}$) [46,47,48]. The Raman signal clearly depended on the concentration of Hb. In the 1500–1650 cm${}^{-1}$ spectral region, the highest Raman intensity at the 1 μg/mL Hb concentration was observed for the NAA substrates without any NPA. The signal from both NPA containing substrates was similar but approximately 50% weaker than the empty NAA case. For the 10 μg/mL solution, the behavior was the opposite, i.e., the Raman intensity progresses from weakest on empty NAA substrates to medium at 60 nm and strongest at 80 nm diameter Au NPA. The surprisingly high Raman signal from low-concentration Hb on an empty NAA in comparison to glass surface (Figure 4b) may be caused by preferred adsorption of Hb on pore surfaces and entrance into the pores [49]. However, for NAA with NPA the signal was less intense since the pores are partly closed with Au nanoparticles. For high-concentration of Hb the NAA surface was most likely saturated and at a 10 times larger concentration the signal from bare NAA (red line in Figure 4c) remained essentially unchanged (only 15% increase relative to Figure 4b). At the same time, the intensity for 80 nm NPA (blue lines in Figure 4b,c) differs approximately 2 times. This indicates that at given Hb concentrations the Au nanoparticle surface is not saturated, which is a useful property for concentration sensing. Due to composition of lyophilized bovine hemoglobin (mixture of predominantly methemoglobin and hemoglobin) the isoelectric point (pI) is not specified but can be assumed to be close to 7.2 [50], which is less than pH 7.4 of the PBS solution. In such conditions electrostatic repulsion between negatively charged Hb molecules can hinder their saturation on Au nanoparticle surface. The ability to use NPAs as SERS sensors is further demonstrated by looking at the spectra taken on pure glass; the Hb Raman signal is very weak, only at the higher concentration the typical Hb Raman bands can be seen, but also then only weakly. The 80 nm Au NPA yield slightly larger sensitivity to Hb concentration in comparison to 60 nm.

## 4. Conclusions

We have demonstrated a new accessible method of plasmonic nanoparticle array production using self-assembly of nanoparticles on self-organized templates. Oxalic acid electrolyte NAA with interpore distances 100 nm was used in combination with 40, 60 and 80 nm Au nanoparticles, which produced NPA with matching geometries. However, the same technique can be adapted for other compatible sets of nanoparticle materials and NAA protocols [27], where pore center separations from 50 nm to over 500 nm are attainable [51]. The Al-NAA-Au NPA multilayers are potentially usable as substrates for both LSPR and SERS-based sensors. The refractometric sensitivity can be tuned by the NPA density, whereas the thickness variation of the NAA membrane allows tuning of scattering peak wavelength in a broad range of the visible spectrum. This is useful, for example, to avoid absorption bands of specific biological macromolecules. For SERS-based sensor substrates the Hb concentration sensitivity at the tested values of 1 and 10 μg/mL increases with NPA particle size. For lower concentration detection the Al-NAA without nanoparticles is an effective substrate; however, it saturates more quickly.
